# Is early age at menarche a risk factor for endometriosis? A systematic review and meta-analysis of case-control studies

**DOI:** 10.1016/j.fertnstert.2012.05.035

**Published:** 2012-09

**Authors:** Kelechi E. Nnoaham, Premila Webster, Jharna Kumbang, Stephen H. Kennedy, Krina T. Zondervan

**Affiliations:** a Department of Public Health, University of Oxford, Oxford, United Kingdom; d Wellcome Trust Centre for Human Genetics, University of Oxford, Oxford, United Kingdom; b Kent Health Protection Unit, Ashford, United Kingdom; c Nuffield Department of Obstetrics and Gynecology, Oxford, United Kingdom

**Keywords:** Endometriosis, menarche, systematic review, meta-analysis

## Abstract

**Objective:**

To review published studies evaluating early menarche and the risk of endometriosis.

**Design:**

Systematic review and meta-analysis of case-control studies.

**Setting:**

None.

**Patient(s):**

Eighteen case-control studies of age at menarche and risk of endometriosis including 3,805 women with endometriosis and 9,526 controls.

**Intervention(s):**

None.

**Main Outcome Measure(s):**

Medline and Embase databases were searched from 1980 to 2011 to locate relevant studies. Results of primary studies were expressed as effect sizes of the difference in mean age at menarche of women with and without endometriosis. Effect sizes were used in random effects meta-analysis.

**Result(s):**

Eighteen of 45 articles retrieved met the inclusion criteria. The pooled effect size in meta-analysis was 0.10 (95% confidence interval −0.01–0.21), and not significantly different from zero (no effect). Results were influenced by substantial heterogeneity between studies (I^2^ = 72.5%), which was eliminated by restricting meta-analysis to studies with more rigorous control of confounders; this increased the pooled effect size to 0.15 (95% confidence interval 0.08–0.22), which was significantly different from zero. This represents a probability of 55% that a woman with endometriosis had earlier menarche than one without endometriosis if both were randomly chosen from a population.

**Conclusion(s):**

There is a small increased risk of endometriosis with early menarche. The potential for disease misclassification in primary studies suggests that this risk could be higher.

**Discuss:** You can discuss this article with its authors and with other ASRM members at http://fertstertforum.com/nnoahamk-early-menarche-endometriosis-systematic-review-meta-analysis/

Endometriosis is characterized by benign proliferation of ectopic endometrial glands and stroma in the peritoneal cavity, resulting in inflammation and scarring, often leading to pelvic pain and infertility (1). It affects 6%–10% of women of reproductive age (2).

The anatomical distribution of endometriotic implants (3) and higher prevalence of the disease in women with obstructive Müllerian anomalies support Sampson's theory of retrograde menstruation as the chief causal mechanism. However, other factors, such as the frequency and volume of menstrual reflux, probably modify the risk. Accordingly, menstrual cycle characteristics (such as age at menarche, shorter menstrual cycle length, and heavy menstruation), which reflect the frequency of exposure to menstruation or volume of menstrual reflux, might be expected to influence endometriosis risk (4, 5).

Early age at menarche, often defined as ≤11 years old (6), might increase a woman's exposure to menstruation during her reproductive lifetime and consequently increase the risk of endometriosis. A number of studies, commonly using case-control designs, have examined the relationship between early age at menarche and endometriosis, with varying conclusions. No specific attempt has been made to review systematically the literature on this possible association.

This systematic review evaluates the association of early age at menarche and risk of endometriosis and combines results of previously published studies in a meta-analysis.

## Materials and methods

### Identification and Selection of Articles

This review was restricted to published research articles that compared age at menarche in women with surgically confirmed endometriosis and those without endometriosis. These studies were identified in two main ways: 1) Medline and Embase databases were searched through the National Library for Health from 1980 to 2011 for all published case-control studies examining the relationship of early age at menarche and the risk of endometriosis. The search was conducted by two of the authors and was limited to human studies published in the English language, and 2) the reference lists of identified publications were also searched in an iterative manner for relevant studies and authors of primary articles contacted where clarity was needed about data in primary studies.

For the database searches, the search terms “case-control studies,” “epidemiologic determinants,” “menarche,” “risk factors,” and “endometriosis” were used as a combination of free text and thesaurus terms (see Supplemental Table 1, available online, for search syntax). Included studies had to 1) be case-control studies involving women with surgically confirmed endometriosis, as the condition can only be diagnosed reliably at surgery, 2) have examined the relationship between endometriosis risk and early age at menarche as a primary or secondary outcome of interest, and 3) have clearly described criteria for the selection of controls.

Important details on design, methods, and results of primary studies were extracted from appropriate articles and summarized.

### Definition of Exposure

Early menarche is often defined as menarche before the age of 12 years (≤11 years old), but some investigators base definition on menarche at ≤12 years. In this review, studies with either definition of early menarche were included.

### Quality of Included Studies

The quality of primary studies was assessed using the Newcastle-Ottawa scale, a validated tool for assessing the quality of observational and nonrandomized studies (7). The scale uses a star system to evaluate observational studies on three criteria: participant selection, comparability of study groups, and assessment of exposure. Key in the assessment of the comparability of study groups is the extent to which potential confounders are controlled for.

### Statistical Analysis

All results were expressed in terms of an “effect size” of the difference in mean age at menarche of women with and without endometriosis. Most studies expressed their findings as odds ratios of early menarche in women with endometriosis compared to controls. For these studies, odds ratios were converted directly to effect sizes using the approach described by Chinn (8). For one study in which results were expressed as the mean ages at menarche in cases and controls, an effect size calculator worksheet was used to derive an effect size from the means and the pooled SDs (9). For another study that expressed the outcome as a median and range, the mean ± SD was estimated using the approach of Hozo et al. (10). Effect sizes were used in random effects meta-analysis of DerSimonian and Laird (11) in Stata program (version 11). The impact of heterogeneity between studies was assessed by calculating the I^2^.

To determine whether any one study unduly influenced the pooled effect size (small study effects), a sensitivity analysis was conducted by recalculating the pooled effect size after deleting each study, one at a time. To explore the presence of publication bias, a funnel plot was produced and the approach by Egger et al. (12) was used to test the significance of funnel plot asymmetry. The latter involves regression of the standard normal deviate of each effect size on the inverse of its standard error (precision). The regression line should have a positive slope and an intercept of zero in the absence of bias.

Further sensitivity and subgroup analyses considered studies by important population and study characteristics. These included: 1) the category of women studied—infertile women versus both fertile and infertile women, 2) the stage of endometriosis studied, 3) the approach to case recruitment—prospective or otherwise, 4) the cutoff age for early age at menarche—≤11 versus ≤12 years, and 5) controlling for important potential confounders, principally body mass index (BMI).

## Results

### Included Studies

Of the 40 studies identified from the database search (list available on request), only 13 fulfilled the predefined entry criteria (see Supplemental Table 2, available online, for excluded studies). Five additional studies were identified from the reference list search. These 18 case-control studies, involving 3,805 cases of surgically diagnosed endometriosis and 9,526 controls, were published between 1986 and 2010 (Table 1) (13–30). The studies were conducted in the United States (n = 4), Italy (n = 4), Canada (n = 2), and the United Kingdom (n = 2); the other six studies were conducted in Australia, Belgium, People's Republic of China, Malaysia, and Spain. Women were generally 18–49 years old, although one study included women up to the age of 69 years (17). The study population in five studies (15, 19, 20, 25, 28) comprised infertile women, although one of those studies (25) used fertile women as controls and another (20) enrolled women who had male factor infertility as controls. All but four studies (14, 20, 22, 30) prospectively recruited women with endometriosis and, although 11 studies defined early age at menarche as <12 years old, in four studies (17–19, 24) it was defined as ≤12 years old. Three studies expressed outcomes as means and medians without defining cutoff ages (21, 23, 30).

### Quality Assessment

**Supplemental Figure 1:**
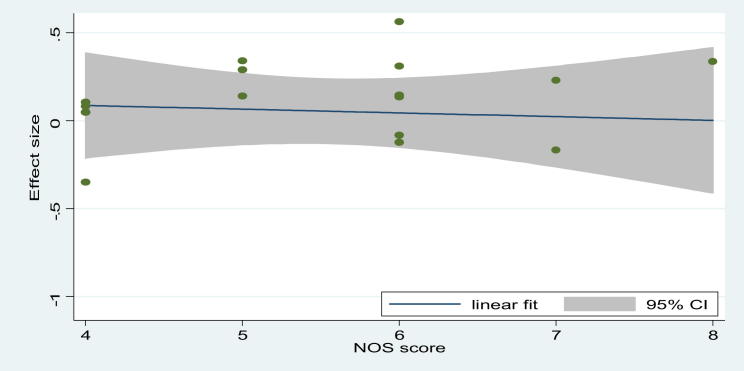
Scatter plot of Newcastle-Ottawa scale (NOS) score and study effect sizes. CI = confidence interval.

The Newcastle–Ottawa quality scores ranged from 4–8 and the mean score for all 18 studies was 5.56 (±SD 1.25). Effect sizes did not significantly vary with quality scores (Supplemental Fig. 1, available online). As shown in Table 2, there was careful selection of cases in included studies, as only surgically confirmed cases were recruited and extent of disease was mostly described in detail. Cases were largely representative of source populations, reducing the risk of selection bias. In two studies, however, patients were reviewed retrospectively for inclusion, with some risk of bias in the case selection (20, 22). Eight studies recruited hospital controls who were either healthy (13) or had diverse conditions such as gynecological diseases (23, 29), acute illnesses (16, 26), trauma (17), infertility (20), and live birth (25). Studies either used community controls (14, 18, 24, 30) or controls sampled from the same cohort as cases (15, 18, 21, 22, 27, 28). However, in only eight of the studies was endometriosis ruled out in controls at laparoscopy (15, 19–22, 27–29). All except two of those eight studies were hospital patient-controlled studies, which sampled controls from the cohort of women undergoing laparoscopy (20, 29).

The overall performance of the included studies on comparability of participants was inadequate. Ten studies (14–17, 20, 24–26, 29, 30) adequately controlled for potential confounders, although only four of those controlled for the potential confounding effect of adult BMI (15, 20, 24, 30). Two studies failed to control for any potential confounders, thereby limiting the comparability of the study groups (18, 21).

The overall performance of the included studies on assessment of exposure was poor. In six of the studies, it was not clear whether exposure ascertainment was blinded (16, 17, 21, 23, 26, 29). Indeed, either the participants or the trained interviewers who collected exposure information in these studies may have been aware of participants' disease status at the time of interview.

### Outcomes

Effect sizes were positive (range, 0.05–0.56) in 13 of the 18 studies (i.e., early menarche associated with greater risk of endometriosis) (13–21, 24–26). Five effect sizes (22, 23, 27–29) were negative (i.e., early menarche associated with reduced risk of endometriosis; range, −1.09 to −0.08). Effect sizes suggested statistically significantly greater risk of endometriosis with early menarche in four studies (18, 20, 21, 30) and significantly lesser risk in two studies (23, 27).

**Figure 1:**
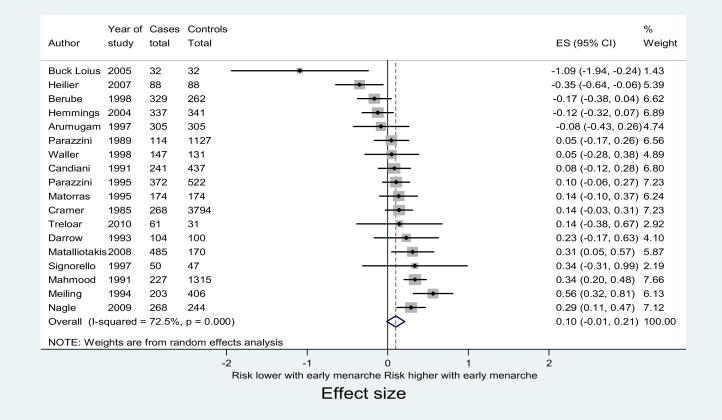
Forest plot of 18 included studies evaluating association between early menarche and endometriosis.

In meta-analysis, a pooled effect size of 0.10 (range, −0.01–0.21) was found (Fig. 1), suggesting that women with endometriosis were 0.10 SDs of age (in years) younger than controls at menarche. This “small” (31) association between early age at menarche and the risk of endometriosis was, however, not statistically significant. An effect of this size, interpreted using the “Common Language Effect Size” approach of McGraw and Wong (32), implies there is a 53% chance that a woman with endometriosis was younger at menarche than a woman without endometriosis if both individuals were chosen at random from a population.

In random effects meta-analysis, a high amount of variation across included studies was explained by heterogeneity rather than chance (χ² = 61.92, *df* = 17; *P*=.000; I² = 72.5%). The effect of this residual heterogeneity on the results was investigated in sensitivity analyses.

### Publication Bias

**Supplemental Figure 2:**
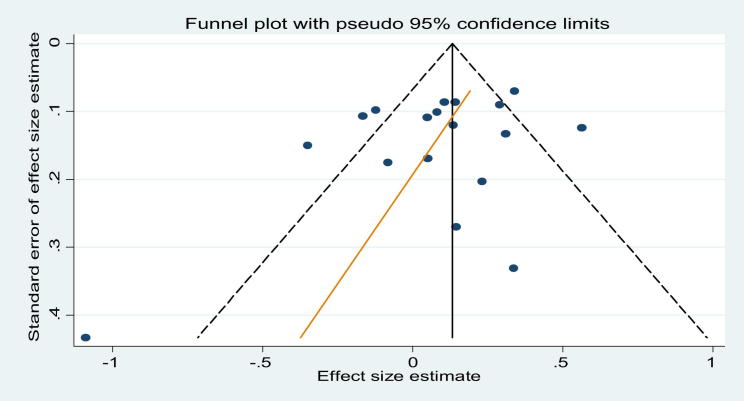
Funnel plot, using data from 18 case-control studies of early menarche and endometriosis risk, with effect sizes displayed on the horizontal axis.

**Supplemental Figure 3:**
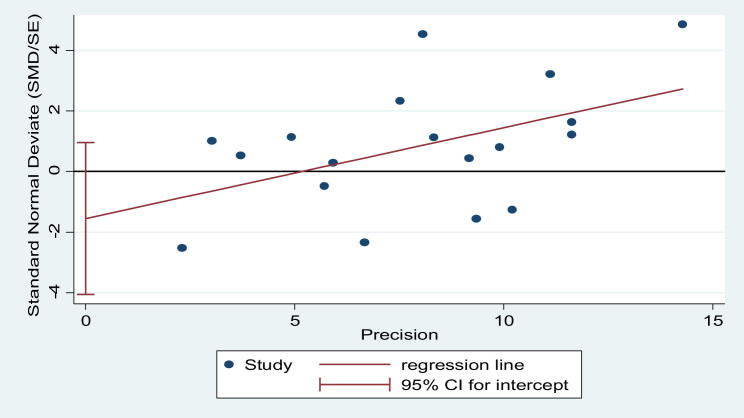
Egger plot. CI = confidence interval.

As shown in the funnel plot in Supplemental Figure 2, visual examination may suggest the presence of funnel plot asymmetry. However, Egger's method to test statistically for the presence of funnel plot asymmetry (Supplemental Fig. 3, available online) shows the regression line to have a positive slope, with no evidence for asymmetry (*t* = −1.31, *P* = .21, 95% confidence interval [CI] −4.06–0.95).

### Sensitivity Analyses

Iterative removal of primary studies from the meta-analysis suggested that two studies (23, 27) with a relatively small sample size may have disproportionately influenced the pooled effect size. After removing each of the other 16 studies, the pooled estimate ranged from 0.05–0.10 and remained nonsignificant. However, after removing these small negative studies, the pooled estimate was 0.15 (95% CI 0.10–0.21).

Other sensitivity analyses were based on a priori stated characteristics of the populations and study designs. Five studies assessed infertile women only (15, 19, 20, 25, 28), although one of those studies (25) compared them to fertile controls. When these five studies only were included in the meta-analysis, residual heterogeneity (measured by the I^2^) decreased to 58% and the summary effect size increased to 0.11 (95% CI −0.06–0.29).

Of the 18 studies included in this review, 2 enrolled only cases with minimal-to-mild endometriosis. Six studies included no information on the disease stage, eight included women with all revised American Fertility Society (AFS) stages, and 2 studies included women with stage III/IV disease. Of the eight studies that enrolled cases of all stages, three predominantly included moderate-to-severe cases (mean, 58% of all cases) and five predominantly included minimal-to-mild cases (mean, 67% of all cases). The pooled effect sizes for the studies with more minimal-to-mild and moderate-to-severe cases were 0.02 (95% CI −0.26–0.29) and 0.32 (95% CI 0.04–0.59), respectively. Heterogeneity remained high in all scenarios within this group of sensitivity analyses.

**Figure 2:**
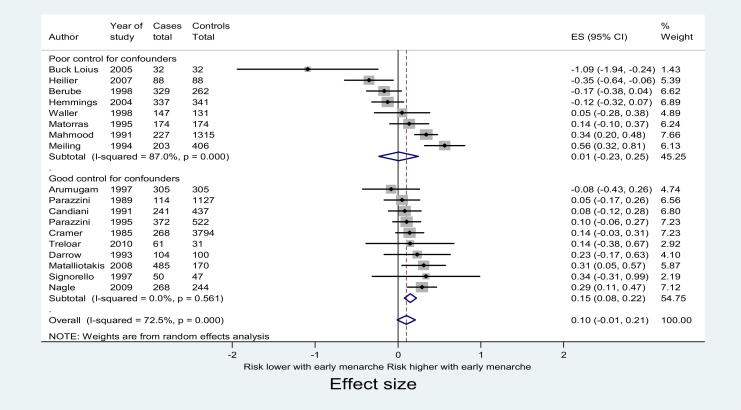
Meta-analysis of included studies presented by rigor of control for potential confounders.

Ten studies adequately controlled for important confounders, thus ensuring comparability of cases and controls (14–17, 20, 24–26, 29, 30). When these studies only were included in the meta-analysis (Fig. 2), the pooled effect size was 0.15 (95% CI 0.08–0.22) and I^2^ was 0, suggesting that the probable source of the variation seen across the 18 included studies was the lack of comparability of cases and controls arising from variation in the adequacy of control for potential confounders.

## Discussion

In this meta-analysis of published case-control studies evaluating the association between age at menarche and endometriosis risk, we found a small, but not statistically significant, increase in risk of endometriosis with early age at menarche (defined as <12 years old). There was substantial heterogeneity across included studies over and above what would be explained by chance alone. Sensitivity analyses suggested that this heterogeneity was explained principally by variations in respect of control of potential confounders of the relationship between age at menarche and endometriosis in individual studies. Consequently, limiting meta-analysis to studies that controlled more rigorously for potential confounders eliminated heterogeneity and suggested that early age at menarche was significantly associated with a higher risk of endometriosis.

Smaller studies are, on average, conducted and analyzed with less methodological rigor than larger studies and trials of lower quality also tend to show the larger effects (12). In this meta-analysis, two small studies (one with a relatively large effect) caused the pooled effect size to tend toward the null value. Their exclusion yielded a larger pooled effect size that suggested that women who were younger at menarche have a significantly higher risk of endometriosis than those who were older.

The inverse relationship between age at menarche and risk of endometriosis was reported previously in a prospective cohort study of fertile and infertile premenopausal women (33). Our study, however, represents the first systematic attempt to review the literature on the relationship between age at menarche and endometriosis risk, and provides a quantitative estimate of the relationship, with careful attention given to understanding the sources of heterogeneity in included primary studies. It uses valid methods of data synthesis that overcome limitations commonly presented by primary studies reporting results as continuous and binary outcomes.

The study highlights the effects that inadequacies in case-control design can have, and has particular relevance to the many other putative risk factors of endometriosis in the literature (5). Well-designed case-control studies of nongenetic risk factors of endometriosis should enroll newly diagnosed cases, collect exposure information predating symptom onset, and use controls representative of the population from which cases are drawn (such as community controls or controls recruited consecutively from the same clinics as cases), with data collected on key confounding factors to allow for adjusted or matched analyses (34). The study of newly diagnosed cases should mitigate the potential bias arising from a change in behavior upon awareness of disease status, although such changes may have occurred already from the time of symptom onset, which in a condition such as endometriosis often precedes diagnosis by many years (35). Collecting information on exposure that predated symptom onset is therefore important, but as such information in case-control studies is collected at the time of diagnosis, differential recall between cases and controls may still produce biased results. It can be argued, however, that this is unlikely to be an important consideration for an exposure such as recall of age at menarche, unless patients are aware of the hypothesis.

The characteristics of the study population in a case-control study of endometriosis are critical to the validity of its findings. To allow generalizability of results, cases should ideally be representative of the general population, but—owing to the lack of a noninvasive diagnostic tool—studies generally recruit as cases women scheduled for laparoscopic investigations to diagnose or rule out endometriosis. As infertility is often a reason for laparoscopy in these women, the frequency of infertile women in a population of cases is artificially raised by this selection mechanism (16). Although this may sufficiently complicate interpretation of findings to warrant studying or analyzing fertile and infertile populations of women with endometriosis separately, the pooled effect size for studies of infertile women only did not differ from the reported pooled effect for other 12 studies (0.11, 95% CI −0.06 to −0.29 vs. 0.09, 95% CI −0.05 to −0.23). Similarly, in the cohort study by Missmer et al. (33), the risk of endometriosis associated with early age at menarche did not significantly differ in infertile women and women without past or concurrent infertility.

It has been suggested that moderate-to-severe, rather than minimal-to-mild endometriosis, represents progressive disease, as the latter may only be a transient phase in an ongoing process that often results in cytolysis of recently implanted endometrial cells (36). We found in this review that on analysis of primary studies of moderate-to-severe disease in exclusion of studies of minimal-to-mild disease, the size and statistical significance of the association between early menarche and endometriosis increased. In light of this finding, we cautiously suggest that early menarche may be associated with the risk of moderate-to-severe, not minimal-to-mild, endometriosis.

Ideally, a case-control study should initially define a source population precisely, from which cases and controls are then randomly sampled. In reality and with specific regard to endometriosis, this means that a source population should be defined explicitly, and should then generate the cases attending for care at a clinic, controls being also sampled randomly from that population. This explicit identification of a source population in endometriosis studies is, however, often unrealistic except in circumstances where a population registry can be compiled. Consequently, in most case-control studies of endometriosis, the source population is defined secondarily to the definition of a case-finding mechanism (e.g., voluntary attendance for care because of symptoms). This secondary definition of a source population on the basis of an identified case series complicates control selection as it is then difficult to demonstrate that controls are members of the same population as cases at the time of sampling. These difficulties notwithstanding, control selection needs to focus on endometriosis-free women who are representative of the population from which cases are drawn. This is especially difficult for endometriosis. Consequently, control women undergoing laparoscopy for sterilization are unlikely to be representative of the symptomatic population from which cases were drawn; indeed community or symptomatic hospital-based controls would be more representative (34). Controls sampled from women with a negative laparoscopy (who are members of the same case series as women who had a positive laparoscopy), would ostensibly be representative of the source population if that population was explicitly defined before case selection, and not secondarily to case series identification. Otherwise, it may be difficult to establish that cases and controls identified through clinics for benign women's health symptoms are representative of the general population in terms of exposure profiles.

Most community- and hospital-based controls in the primary studies in this review did not have endometriosis ruled out by laparoscopy, raising the possibility of disease misclassification. Furthermore, hospital-based controls should ideally not have conditions related to the exposure of interest. In one study (29), some hospital controls had ovarian tumors, which have been linked positively with early menarche (37). Misclassification and use of controls with exposure-related conditions also potentially alter the relationship between age at menarche and endometriosis risk.

In addition to other important potential confounders, such as age and socioeconomic status, adult BMI confounds the relationship between early age at menarche and endometriosis risk, being inversely related to both early age at menarche and the risk of endometriosis (38). Only 10 of the 18 studies adequately controlled for potential confounders. As indicated by the results of the sensitivity analyses, residual heterogeneity was due largely to the inclusion of studies with less rigorous control of confounding.

The potential for misclassification of disease in the primary studies means that the actual pooled effect size found in our meta-analysis ought to be viewed with some caution. All cases were diagnosed through laparoscopy, which may not be fail proof as evidenced by the reported intraobserver and interobserver agreements for visualization of endometriotic lesions during the procedure (39). The presence of disease misclassification would, however, have underestimated the relationship between age at menarche and endometriosis risk. Furthermore, age at menarche was self-reported in most included studies but the validity of age at menarche self-reported in middle age is only moderate compared with that recorded in adolescence (40). The impact of potential recall bias is, however, unlikely to be significant as there is no evidence to suggest that recall might be differential between cases and controls. It should be noted that, although this review provides a quantitative measure of the relationship between early age at menarche and endometriosis risk, the pooled effect size, being a weighted standardized mean difference, may be more clinically meaningful if directly interpreted qualitatively, rather than quantitatively.

This review concludes that early age at menarche is associated with a very modest increase in endometriosis risk when studies with better methodological quality adequate control of potential confounders are considered. It highlights the 1) need for well-designed studies incorporating collection of confounder information to explore other risk-factors that may be even more subject to bias, and 2) the need to understand the significance of these factors in the diagnosis of endometriosis and understanding of its etiology. Finally, it has been suggested that a history of earlier age at menarche may be used to guide diagnostic and therapeutic strategies if other symptoms point to endometriosis as a possible diagnosis (14). The results of this meta-analysis, however, do not present strong evidence for the clinical utility of a history of early menarche in the evaluation of endometriosis.

## Figures and Tables

**Supplemental Table 1:** Syntax for search strategy in Medline.

Search term
MENARCHE/
menarche.ti,ab
1 OR 2
ENDOMETRIOSIS/
endometriosis.ti,ab
4 OR 5
3 AND 6
RISK FACTORS/
risk*.ti
(“risk factor*” OR determinant*).ti,ab
epidemiolog*.ti
8 OR 9 OR 10 OR 11
6 AND 12
ENDOMETRIOSIS/ep,et [ep=Epidemiology, et=Etiology]
7 OR 13 OR 14
exp CASE-CONTROL STUDIES/
(case* AND control*).ti,ab
16 OR 17
15 AND 18
19 [Limit to: Publication Year 1980–2011 and English Language]

**Supplemental Table 2:** Studies not included and reasons for noninclusion.

S/N	Authors	Title	Journal/year/volume	Reason for noninclusion
1	Mamdouh HM; Mortada MM; Kharboush IF; Abd-Elateef HA	Epidemiologic determinants of endometriosis among Egyptian women: a hospital-based case-control study	*Journal of the Egyptian Public Health Association*/2011/86	Did not evaluate risk of endometriosis associated with early menarche
2	Bellelis P; Dias JA Jr.; Podgaec S; Gonzales M; Baracat EC; Abrao MS	Epidemiologic and clinical aspects of pelvic endometriosis—a case series	*Revista Da Associacao Medica Brasileira*/2010/56	Not a case-control study
3	Nouri K; Ott J; Krupitz B; Huber JC; Wenzl R	Family incidence of endometriosis in first-, second-, and third-degree relatives: case-control study	*Reproductive Biology & Endocrinology*/2010/8	Did not evaluate risk of endometriosis associated with early menarche
4	Zhu Z; Al-Beiti MA; Tang L; Liu X; Lu X	Clinical characteristic analysis of 32 patients with abdominal incision endometriosis	*Journal of Obstetrics & Gynaecology*/2008/28	Did not evaluate risk of endometriosis associated with early menarche
5	Matalliotakis IM; Arici A; Cakmak H; Goumenou AG; Koumantakis G; Mahutte NG	Familial aggregation of endometriosis in the Yale series	*Archives of Gynecology & Obstetrics*/2008/278	Age at menarche compared between women with “endometriosis + family history” vs. “endometriosis no family history”
6	Parazzini F; Cipriani S; Bianchi S; Gotsch F; Zanconato G; Fedele L	Risk factors for deep endometriosis: a comparison with pelvic and ovarian endometriosis	*Fertility & Sterility*/2008/90	Multiple case groups complicating comparison
7	Sinaii N; Plumb K; Cotton L; Lambert A; Kennedy S; Zondervan K; Stratton P	Differences in characteristics among 1,000 women with endometriosis based on extent of disease	*Fertility & Sterility*/2008/89	Did not evaluate risk of endometriosis associated with early menarche
8	Kvaskoff M; Mesrine S; Clavel-Chapelon F; Boutron-Ruault MC	Endometriosis risk in relation to naevi, freckles, and skin sensitivity to sun exposure: the French E3N cohort	*International Journal of Epidemiology*/2009/38	Did not evaluate risk of endometriosis associated with early menarche
9	Hediger ML; Hartnett HJ; Louis GM	Association of endometriosis with body size and figure	*Fertility & Sterility*/2005/84	Very small sample size
10	Modugno F; Ness RB; Allen GO; Schildkraut JM; Davis FG; Goodman MT	Oral contraceptive use, reproductive history, and risk of epithelial ovarian cancer in women with and without endometriosis	*American Journal of Obstetrics & Gynecology*/2004/191	Did not evaluate risk of endometriosis associated with early menarche
11	Parazzini F; Chiaffarino F; Surace M; Chatenoud L; Cipriani S; Chiantera V; Benzi G; Fedele L	Selected food intake and risk of endometriosis	*Human Reproduction*/2004/19	Did not evaluate risk of endometriosis associatedwith early menarche
12	Meaddough EL; Olive DL; Gallup P; Perlin M; Kliman HJ	Sexual activity, orgasm, and tampon use are associated with a decreased risk for endometriosis	*Gynecologic & Obstetric Investigation*/2002/53	Cases were not reported to have surgically confirmed endometriosis
13	Cramer DW; Missmer SA	The epidemiology of endometriosis	*Annals of the New York Academy of Sciences*/2002/955	Not a case-control study
14	Cahill DJ; Hull MG	Pituitary-ovarian dysfunction and endometriosis	*Human Reproduction Update*/January/6	Did not evaluate risk of endometriosis associated with early menarche
15	Laufer MR; Goitein L; Bush M; Cramer DW; Emans SJ	Prevalence of endometriosis in adolescent girls with chronic pelvic pain not responding to conventional therapy	*Journal of Pediatric & Adolescent Gynecology*/1997/10	Did not evaluate risk of endometriosis associated with early menarche
16	Moen MH; Schei B	Epidemiology of endometriosis in a Norwegian county	*Acta Obstetricia et Gynecologica Scandinavica*/1997/76	Case group likely to have included women without surgically confirmed endometriosis
17	Eskenazi B; Warner ML	Epidemiology of endometriosis	*Obstetrics & Gynecology Clinics of North America*/1997/24	Evidence summary
18	Reese KA; Reddy S; Rock JA	Endometriosis in an adolescent population: the Emory experience	*Journal of Pediatric & Adolescent Gynecology*/1996/9	Retrospective case review
19	Sangi-Haghpeykar H; Poindexter AN	Epidemiology of endometriosis among parous women	*Obstetrics & Gynecology*/1995/85	Did not evaluate risk of endometriosis associated with early menarche
20	Han M; Pan L; Wu B; Bian X	A case-control epidemiologic study of endometriosis	*Chinese Medical Sciences Journal*/1994/9	Did not evaluate early menarche and risk of endometriosis as primary or secondary outcome of interest
21	Darrow SL; Selman S; Batt RE; Zielezny MA; Vena JE	Sexual activity, contraception, and reproductive factors in predicting endometriosis	*American Journal of Epidemiology*/1994/140	Did not evaluate risk of endometriosis associated with early menarche
22	Parazzini F; Ferraroni M; Bocciolone L; Tozzi L; Rubessa S; La Vecchia C	Contraceptive methods and risk of pelvic endometriosis	*Contraception*/1994/49	Did not evaluate risk of endometriosis associated with early menarche
23	Moen MH; Magnus P	The familial risk of endometriosis	*Acta Obstetricia et Gynecologica Scandinavica*/1993/72	Did not evaluate risk of endometriosis associated with early menarche
24	McCann SE; Freudenheim JL; Darrow SL; Batt RE; Zielezny MA	Endometriosis and body fat distribution	*Obstetrics & Gynecology*/1993/82	Risk of endometriosis associated with early menarche was not a primary or secondary outcome
25	Parazzini F; Ferraroni M	Epidemiology of endometriosis	*BMJ*/1993/306	Not a case-control study
26	Kirshon B; Poindexter AN	Contraception: a risk factor for endometriosis	*Obstetrics & Gynecology*/1988/71	Risk of endometriosis associated with early menarche was not a primary or secondary outcome
27	Makhlouf Obermeyer C; Armenian HK; Azoury R	Endometriosis in Lebanon. A case-control study	*American Journal of Epidemiology*/1986/124	Did not evaluate risk of endometriosis associated with early menarche

**Table 1:** Summary of included studies.

Author, year, place	Study population	Study design	Cases	Controls	Parameter measured	Result	Reviewer's comments
Arumugam 1997, Malaysia	Women aged 19–45 years, admitted to gynecology wards in two hospitals and undergoing laparoscopy or laparotomy	Case-control	305 prospectively enrolled women with laparoscopically diagnosed endometriosis	305 age-matched hospital controls with fibroids, ovarian tumors, EP, DUB, pelvic inflammatory disease, and infertility	Odds of endometriosis in women <12 years at menarche compared with those ≥12 years at menarche	OR 0.86 (95% CI 0.42–1.45)	Although controls had endometriosis surgically ruled out, they had other gynecological indications for surgery
Berube 1998, Canada	Women aged 20–39 years, infertile, undergoing diagnostic laparoscopy	Case-control	329 prospectively enrolled cases with laparoscopically diagnosed minimal and mild endometriosis	262 controls were women (from same cohort) who did not have endometriosis on laparoscopy	Odds of endometriosis in women <12 years at menarche compared with those ≥12 years at menarche	OR 0.74 (95% CI 0.51–1.08)	Compared infertile cases to infertile controls and women had no other known factors explaining their infertility other than endometriosis in cases
Buck Louis 2005, USA	Women aged 18–40 years and scheduled for laparoscopy for suspected endometriosis, infertility, pelvic pain, tubal ligation, pelvic inflammatory disease, polycystic ovaries, or fibroids	Case-control	32 prospectively enrolled women with laparoscopically diagnosed endometriosis	52 women (from same cohort) without endometriosis	Odds of endometriosis in women <12 years at menarche compared with those ≥12 years at menarche	OR 0.14 (95% CI 0.03–0.65)	
Candiani 1991, Italy	Women aged 20–49 years, attending different hospitals	Case-control	241 prospectively enrolled cases with infertility, pelvic pain, or pelvic masses, and laparoscopically diagnosed endometriosis	437 hospital controls with acute conditions, attending hospitals near the one from which cases were recruited	Odds of endometriosis in women <12 years at menarche compared with those ≥12 years at menarche	OR 1.16 (95% CI 0.81–1.66)	No specific work-up done in controls to rule out endometriosis
Cramer 1986, USA	Infertile women constituted cases whereas controls were fertile women	Case-control	268 prospectively enrolled cases with infertility and laparoscopically diagnosed endometriosis	3,794 hospital controls were fertile women who had just delivered live-born infants at the same hospital	Odds of endometriosis in women <12 years at menarche compared with those ≥12 years at menarche	OR 1.29 (95% CI 0.95–1.75)	Fertile women were used as controls for infertile women, with potential for bias. Furthermore, in fertile women, endometriosis was not ruled out by laparoscopy. Adjusted for age, center, religion, and education
Darrow 1993, USA	Women aged 19–45 years attending hospital for laparoscopy, and their friends	Case-control	104 prospectively enrolled cases with laparoscopically diagnosed endometriosis	100 friend controls	Odds of endometriosis in women ≤12 years at menarche compared with those >12 years at menarche	OR 1.52 (95% CI 0.74–3.13)	Friend controls were only screened for endometriosis using a questionnaire. Medical controls also used. Medical controls underestimated risks
Heilier 2007, Belgium	Women attending gynecology clinics for various reasons	Case-control	88 prospectively enrolled cases of laparoscopically diagnosed peritoneal endometriosis	88 age-matched hospital controls, without complaints of infertility, pelvic pain, or dysmenorrhea	Median and range of age at menarche for cases compared with controls	Cases: median 13 years (range, 9–18 y); controls: median 12.5 years (range, 9–17 y)	Controls were not excluded from endometriosis through laparoscopy but by pelvic examination
Hemmings 2004, USA	Cohort of women scheduled to undergo laparoscopy or laparotomy	Case-control	337 retrospectively enrolled women diagnosed with endometriosis on laparoscopy	341 controls (from same cohort) who did not have endometriosis on laparoscopy	Odds of endometriosis in women <12 years at menarche compared with those ≥12 years at menarche	OR 0.80 (95% CI 0.6–1.2)	
Mahmood 1991, UK	Women scheduled for laparoscopy for infertility, tubal sterilization or chronic pelvic pain, and women scheduled for total abdominal hysterectomy for DUB	Case-control	227 prospectively enrolled cases of laparoscopically diagnosed endometriosis	1,315 controls (from same cohort) who did not have endometriosis on laparoscopy	Mean and SD of age at menarche for cases and controls	Cases: mean 12.54 years (SD 1.53 y); controls: mean 13.07 years (SD 1.58 y)	
Matalliotakis 2008, USA	Infertile women cared for in a hospital within preceding 6 years of the study	Case-control	485 retrospectively enrolled women with pelvic pain and infertility and laparoscopically diagnosed endometriosis	170 hospital controls surgically confirmed not to have endometriosis; infertile women	Odds of endometriosis in women <12 years at menarche compared with those ≥12 years at menarche	OR 1.76 (95% CI 1.10–2.83)	Cases not prospectively enrolled. Source of controls not very clearly stated, although it appeared that they were also infertile patients from same hospital
Matorras 1995, Spain	Infertile women scheduled for laparoscopy	Case-control	174 prospectively enrolled cases with laparoscopically diagnosed endometriosis	174 controls (from same cohort) who did not have endometriosis on laparoscopy	Odds of endometriosis in women ≤12 years at menarche compared with those >12 years at menarche	OR 1.28 (95% CI 0.84–1.97)	Compared infertile cases to infertile controls
Meiling 1994, People's Republic of China	Women <45 years with laparoscopically confirmed endometriosis and population controls	Case-control	203 prospectively enrolled cases with laparoscopically diagnosed endometriosis; no specified population	406 community controls selected from the same residential area as patients	Odds of endometriosis in women ≤12 years at menarche compared with those >12 years at menarche	OR 2.77 (95% CI 1.78–4.29)	Symptomless controls selected from same source population as patients and had careful pelvic examination and ultrasonography to rule out pathology
Nagle 2009, Australia	Women aged 18–55 years recruited from a genetic study of endometriosis and the Australian Twin Registry	Case-control	268 women with laparoscopically diagnosed moderate/severe endometriosis	244 women selected from twin pairs enrolled with the Australian Twin Registry matched to cases on age and geographic location	Mean and SD of age at menarche for cases and controls	Cases: mean 12.6 years (SD 1.4 y); controls: mean 13.0 years (SD 1.4 y)	Cases and controls selected from different catchment populations. Furthermore, unclear how endometriosis was excluded in controls since they were sampled from enrollees in Twin Registry
Parazzini 1989, Italy	20- to 69-year-old women admitted to hospital for histologically confirmed ovarian cysts	Case-control	114 prospectively enrolled cases with histologically confirmed endometrioid ovarian cysts	1,127 hospital controls admitted mainly for trauma	Odds of endometriosis in women ≤12 years at menarche compared with those >12 years at menarche	OR 1.09 (95% CI 0.74–1.6)	Excluded women with gynecological, hormonal, or neoplastic diseases from controls
Parazzini 1995, Italy	Women aged 20–49 years, attending different hospitals	Case-control	372 prospectively enrolled cases with infertility, pelvic pain, or pelvic masses, and laparoscopically diagnosed endometriosis	522 hospital controls with acute conditions, attending hospitals near the one from which cases were recruited	Odds of endometriosis in women <12 years at menarche compared with those ≥12 years at menarche	OR 1.21 (95% CI 0.89–1.64)	Cases and controls selected from different catchment populations. Excluded women with gynecological, hormonal, or neoplastic diseases from controls
Signorello 1997, Italy	Infertile women aged 23–44 years, scheduled for laparoscopy	Case-control	50 prospectively enrolled cases; infertile women with laparoscopically diagnosed endometriosis	47 infertile women (from same cohort) without endometriosis	Odds of endometriosis in women <12 years at menarche compared with those ≥12 years at menarche	OR 1.84 (95% CI 0.57–5.97)	Compared infertile cases to infertile controls
Treloar 2010, Australia	Women aged 18–55 years recruited from a genetic study of endometriosis and the Australian Twin Registry	Case-control	61 cases; women with laparoscopically diagnosed moderate/severe endometriosis	31 women without endometriosis age-matched to cases	Odds of endometriosis in women <12 years at menarche compared with those ≥12 years at menarche	OR 1.3 (95% CI 0.5–3.4)	Cases and controls selected from different catchment populations
Waller 1998, UK	Women with laparoscopically confirmed endometriosis and hospital controls	Case-control	147 prospectively and retrospectively recruited women with laparoscopically diagnosed endometriosis	131 hospital controls (healthy women attending well women or family planning clinics for routine cytology or advice about starting or restarting contraception)	Odds of endometriosis in women <12 years at menarche compared with those ≥12 years at menarche	OR 1.10 (95% CI 0.6–2.0)	

*Note:* 95% CI = 95% confidence interval; DUB = dysfunctional uterine bleeding; EP = ectopic pregnancy; OR = odds ratio.

**Table 2:** Quality of included studies using Newcastle-Ottawa scale.

Author	Selection				Comparability		Exposure			Score
Arumugam 1997										6
Berube 1998										7
Buck Louis 2005										7
Candiani 1991										4
Cramer 1986										5
Darrow 1993										7
Heilier 2007										4
Hemmings 2004										6
Mahmood 1991										5
Matalliotakis 2008										6
Matorras 1995										6
Meiling 1994										6
Nagle 2009										5
Parazzini 1989										4
Parazzini 1995										4
Signorello 1997										8
Treloar 2010										6
Waller 1998										4
